# A hybrid machine learning model combining association rule mining and classification algorithms to predict differentiated thyroid cancer recurrence

**DOI:** 10.3389/fmed.2024.1461372

**Published:** 2024-10-04

**Authors:** Feyza Firat Atay, Fatma Hilal Yagin, Cemil Colak, Emin Tamer Elkiran, Nasrin Mansuri, Fuzail Ahmad, Luca Paolo Ardigò

**Affiliations:** ^1^Department of Internal Medicine and Medical Oncology, Faculty of Medicine, Inonu University, Malatya, Turkey; ^2^Department of Biostatistics and Medical Informatics, Faculty of Medicine, Inonu University, Malatya, Turkey; ^3^Clinical Laboratory Sciences, College of Applied Medical Sciences, King Khalid University, Abha, Saudi Arabia; ^4^Department of Respiratory Care, College of Applied Sciences, Almaarefa University, Diriya, Riyadh, Saudi Arabia; ^5^Department of Teacher Education, NLA University College, Oslo, Norway

**Keywords:** differentiated thyroid cancer, recurrence prediction, associative classification, machine learning, personalized medicine

## Abstract

**Background:**

Differentiated thyroid cancer (DTC) is the most prevalent endocrine malignancy with a recurrence rate of about 20%, necessitating better predictive methods for patient management. This study aims to create a relational classification model to predict DTC recurrence by integrating clinical, pathological, and follow-up data.

**Methods:**

The balanced dataset comprises 550 DTC samples collected over 15 years, featuring 13 clinicopathological variables. To address the class imbalance in recurrence status, the Synthetic Minority Over-sampling Technique for Nominal and Continuous (SMOTE-NC) was utilized. A hybrid model combining classification algorithms with association rule mining was developed. Two relational classification approaches, regularized class association rules (RCAR) and classification based on association rules (CBAR), were implemented. Binomial logistic regression analyzed independent predictors of recurrence. Model performance was assessed through accuracy, sensitivity, specificity, positive predictive value, negative predictive value, and F1 score.

**Results:**

The RCAR model demonstrated superior performance over the CBAR model, achieving accuracy, sensitivity, and F1 score of 96.7%, 93.1%, and 96.7%, respectively. Association rules highlighted that papillary pathology with an incomplete response strongly predicted recurrence. The combination of incomplete response and lymphadenopathy was also a significant predictor. Conversely, the absence of adenopathy and complete response to treatment were linked to freedom from recurrence. Incomplete structural response was identified as a critical predictor of recurrence risk, even with other low-recurrence conditions.

**Conclusion:**

This study introduces a robust and interpretable predictive model that enhances personalized medicine in thyroid cancer care. The model effectively identifies high-risk individuals, allowing for tailored follow-up strategies that could improve patient outcomes and optimize resource allocation in DTC management.

## 1 Introduction

Among the diverse array of endocrine malignancies, differentiated thyroid carcinoma (DTC) stands out as the most prevalent, presenting a unique challenge within the realm of oncology due to its variable rates of recurrence. These fluctuating recurrence patterns can profoundly influence patient management strategies and long-term outcomes, underscoring the complexity of this disease (1). Thyroid cancer is becoming more common, especially DTC. The degree of differentiation of thyroid carcinomas at the DTC and undifferentiated (anaplastic) ends of the illness spectrum has been used to classify these tumors. There is a clear difference in the morphology and behavior of these two species. Papillary and follicular carcinomas are the two types of DTCs. The undifferentiated group includes anaplastic, insular, and other forms of carcinoma at the other end of the range. In terms of behavior, anaplastic carcinomas are extremely aggressive, whereas papillary and follicular carcinomas are generally mild and treatable (2).

According to SEER (Surveillance, Epidemiology, and End Results Program) data, the estimated number of cases in 2024 is 44,020. This rate constitutes 2.2% of all cancer cases in 2024. The estimated number of deaths in 2024 is 2,170. However, the overall 5-year survival rate for thyroid cancer is quite good. While the 5-year expected survival for localized disease is 99%, it drops to 51% for distant metastasis. It typically peaks between the ages of 55 and 64. The papillary type, especially after increasing in 2014–2015, has shown a decreasing trend in the thyroid, whereas the follicular type has tended to remain stable for over twenty years. We detect the vast majority of patients while they are still localized (3).

Although the etiology of thyroid cancers is not clearly known, many factors have been blamed, especially DTCs found in endemic goiter regions and people exposed to radiation in childhood (4). Despite the generally favorable prognosis associated with DTC, the disease exhibits a notable propensity for recurrence in approximately 20% of patients, highlighting the critical need for the development of superior predictive methodologies. Such methodologies would enable the identification of high-risk individuals and facilitate the tailoring of treatment regimens, ultimately optimizing patient outcomes (5).

Recent years have witnessed remarkable advancements in the fields of data mining and machine learning, ushering in novel avenues for enhancing the accuracy of recurrence predictions across various medical domains. Amidst these innovative approaches, associative classification has emerged as a particularly promising technique, demonstrating its potential in diverse medical applications (6). This methodology synergistically amalgamates the strengths of association rule mining with the predictive prowess of classification algorithms. This method uses the pattern recognition features of association rule mining to find complex patterns and relationships in very large datasets, patterns that might be missed by other methods. Consequently, associative classification promises to deliver more accurate and robust predictions, a critical asset in the medical realm.

Regarding thyroid cancer, the application of associative classification could potentially reveal unique combinations of clinical and pathological features that exhibit heightened predictive power for recurrence events. Artificial intelligence/machine learning (AI/ML) models have demonstrated considerable potential in forecasting outcomes for different forms of cancer. These models can combine and merge many types of data, such as genetic, histological, and clinical data, to deliver precise prognostic information (7). However, the specific application of associative classification in predicting DTC recurrence remains an underexplored domain, representing fertile ground for further investigation.

Regularized Class Association Rules (RCAR) is an advanced data mining technique designed for class-imbalanced datasets that enhance predictive accuracy and model simplicity by generating concise, regularized rules that directly link feature sets to class labels, preventing overfitting and improving interpretability (8–10). The Classification Based on Association Rules (CBAR) method combines association rule mining with classification to create an understandable model. It does this by creating and prioritizing relevant rules based on confidence, support, and lift. This makes predictions clear and accurate, especially in fields where it’s important to know why decisions are made (8, 11, 12). Both RCAR and CBAR use association rules to classify, but RCAR uses regularization to deal with class imbalance and overfitting, while CBAR focuses on accuracy and readability without specifically dealing with class imbalance or model complexity. Which one to use depends on the dataset requirements and the desired balance between accuracy and simplicity (8).

The aim of this study is to develop and evaluate an associative classification model that integrates clinical, pathological, and follow-up data to accurately predict the recurrence of DTC. This model aims to personalize follow-up strategies based on individual risk profiles by enhancing predictive accuracy, improving patient outcomes, optimizing resource allocation, and advancing personalized medicine in thyroid cancer care. This approach seeks to identify high-risk individuals for intensive monitoring or adjuvant therapies while reducing unnecessary follow-up for low-risk patients, thereby contributing to the overall improvement of DTC management and prognosis in the broader context of cancer care (13).

## 2 Materials and methods

### 2.1 Dataset explanation

The open-access dataset employed in this study represents a rich and meticulously curated repository of clinicopathologic data, encompassing a comprehensive array of 13 features spanning a substantial period of 15 years. The main objective of the relevant dataset is to facilitate the prediction of recurrence in patients diagnosed with DTC, a malignancy characterized by its variable prognoses and treatment outcomes. Notably, each patient included in the dataset was followed for a minimum of 10 years, ensuring a longitudinal perspective and enabling rigorous analyses of long-term outcomes. This dataset was meticulously compiled with the express purpose of advancing research at the intersection of artificial intelligence and medical sciences, with a particular emphasis on elucidating the factors contributing to the recurrence of thyroid cancer. The dataset encompasses a diverse array of features, encompassing both categorical and numerical variables, thereby enabling a multidimensional approach to understanding the predictors of cancer recurrence. These features include demographic characteristics such as age and gender, clinicopathologic parameters like histologic code, capsule invasion, and lymph node involvement, as well as treatment-related variables such as surgical procedure, tumor size, and radiation therapy. This multifaceted representation of patient data provides a holistic method for exploring the intricate interplay between various factors and their potential impact on cancer recurrence. The balanced dataset using the relevant technique in the current study comprises 550 instances, each representing a unique patient diagnosed with well-DTC. The target variable is a binary indicator that delineates whether the cancer recurred [coded as 1] or did not recur [coded as 0] during the follow-up period. The remaining features encompass a comprehensive array of clinically relevant parameters, including age, gender, histologic code, capsule invasion, extension beyond the thyroid capsule, lymph node involvement, multifocal tumor, extracapsular extension, surgical procedure, tumor size, radiation treatment, and metastasis (14). The Inonu University Health Sciences Non-Interventional Clinical Research Ethics Committee approved this study (approval number: 2024/6061).

### 2.2 Patient stratification, diagnosis and treatment

Patient data were stratified as follows: patient’s age at diagnosis, sex (male or female), current smoking status, past smoking history, presence of goiter (diffuse, single nodular goiter in the left or right lobe, multinodular or normal), presence of adenopathy on physical examination (absent, anterior right, anterior left, bilateral, bilateral, posterior or diffuse including all previously mentioned sites), thyroid function (classified as euthyroid, clinical or subclinical hypo/hyperthyroidism), focality (unifocal, multifocal) and risk assessment according to ATA guidelines (low, intermediate and high), Initial treatment response (good, biochemically incomplete, structurally incomplete, indeterminate), TNM staging (individual T, N, and M scores and final stage) history of radiation therapy to the head and neck region, and recurrence status (including both locoregional and distant metastases) are considered. Per the 2015 revisions of “Treatment Guidelines for Patients with Thyroid Nodules and Differentiated Thyroid Cancer” by the American Thyroid Association, the classification of thyroid tumors is based on the degree of risk as high, intermediate, and low (15). Patients who were over 18 years of age, diagnosed with differentiated thyroid cancer, who continued their follow-up and underwent surgery were included in the study. Exclusion criteria were defined as patients who were not surgical candidates, dedifferentiated, and who did not follow-up after surgery. Scintigraphy is one of the most commonly used preoperative diagnostic methods for the diagnosis of thyroid cancer. It should be kept in mind that patients diagnosed with multinodular goiter or diffuse goiter on thyroid scintigraphy have a possibility of thyroid cancer. Currently, preoperative characterization of lesions can be best performed by fine needle aspiration biopsy. With this method, the need for surgery and the type of surgery to be performed are decided. Computed tomography is recognized as the most effective method for demonstrating metastasis and invasion of surrounding tissue. Invasion of regional vessels and nerves must be detected preoperatively (16). Treatment options for thyroid cancer are divided into three main groups: surgery, chemotherapy, and radiotherapy. Effective and accurate use of preoperative diagnostic methods is recommended for treatment planning. Surgery is considered the most appropriate option in patients who are suitable for surgery and who have not metastasized (17).

### 2.3 Data collection and quality

The dataset was meticulously curated, ensuring no missing values and capturing a comprehensive array of demographic, historical, and clinical data. This level of detail makes predictive models for thyroid cancer recurrence much more reliable and robust. It also lets us look into the complex relationships between clinicopathologic factors and outcomes in more depth. The high data quality reflects the study’s commitment to scientific rigor and lays a strong foundation for advancing medical research in this field (1, 5).

### 2.4 Data preprocessing and development of predictive models

In this study, the Synthetic Minority Oversampling Technique for Nominal and Continuous Data (SMOTE-NC) was employed to address class imbalance by generating synthetic examples for underrepresented classes, accommodating both categorical and continuous features. This data preprocessing step was followed by the development of an associative classification model, a hybrid machine learning approach that combines classification algorithms with association rule mining (6, 18, 19). This method allows for the detection of complex, non-linear relationships between variables, making it particularly effective for predictive modeling in medical contexts. The associative classification model is designed to produce interpretable predictions by using high-quality association rules, which enhances its ability to capture intricate patterns linked to thyroid cancer recurrence. The model’s performance is evaluated using metrics such as accuracy, sensitivity, specificity, positive predictive value, negative predictive value, and F1 score (20, 21). Additionally, the current study explored the use of RCAR, a technique that incorporates regularization to manage class imbalance and prevent overfitting, ensuring a balance between model simplicity and predictive accuracy. The CBAR method was also considered, focusing on leveraging association rules for high interpretability and accuracy, making it particularly valuable in domains where understanding the rationale behind predictions is crucial. The choice between RCAR and CBAR depends on the specific needs of the dataset and the desired trade-off between accuracy and model simplicity (13).

### 2.5 Statistical analysis

Both analytical (Shapiro-Wilk Test) and visual (histogram and probability plots) techniques were used to assess the variables’ conformance to the normal distribution. The Levene test was used to investigate the homogeneity of variances assumption. When it comes to quantitative data with a normal distribution, descriptive statistics are expressed as mean (standard deviation). To compare two unrelated groups based on variables that matched the parametric test assumptions, the independent samples t-test was employed. Categorical data were summarized with frequency and percentage, and the chi-square test was used for group comparison. Binomial logistic regression analysis was used in the multivariate analysis to examine independent predictors for thyroid cancer recurrence. The advanced feature selection strategy was followed when doing the logistic regression analysis. The logistic regression model and its coefficients were assessed using the Hosmer-Lemeshow and Omnibus tests. A p-value of less than 0.05 was deemed statistically significant for all results. Analyses were performed using Python 3.9 software and IBM SPSS Statistics 28.0 (IBM Corp., Armonk, NY, United States) package program.

## 3 Results

The demographic data shown in Table 1 elucidates notable differences across patient groups based on cancer recurrence. The group that did not experience a recurrence had an average age of 38.41 years (SD = 12.94), while the group that experienced one had a substantially higher mean age of 46.698 years (SD = 17.95), with a p-value of less than 0.001. In terms of gender distribution, there were more females in the group without recurrence (57.5%) than in the group with recurrence (42.5%). On the other hand, males consisted of 76.2% of the recurrent group and 23.8% of the non-recurrent group. With a p-value of less than 0.001, the gender distribution difference was statistically significant (Table 1).

**TABLE 1:** Descriptive statistics in the demographic information across the groups.

Variable		Recurred		*p*-value
		No (*n* = 275)	Yes (*n* = 275)	
Age*		38.41 (12.94)	46.698 (17.95)	<0.001
Gender**	Female	246 (57.5)	182 (42.5)	<0.001
	Male	29 (23.8)	93 (76.2)	

*Variable was expressed as mean (standard deviation) and a t-test was used in independent groups for the group comparison.

**Variable was expressed as frequency (percentage) and chi-square test was used for group comparison.

Table 2 presents the statistical analysis results of the variables between the groups. The groups with and without recurrence differed significantly across several factors, according to the statistical analysis. The recurrent group had a higher frequency of smoking (81.8%) than the non-recurrent group (18.2%), with a p-value of less than 0.001. There was a significant difference in thyroid function; most recurred patients (53.1%) were euthyroid, whereas non-recurred cases were less frequently euthyroid (46.9%). Physical examination revealed that only the non-recurrent group (100%) had diffuse goiter, while the recurrent group had a greater incidence of multinodular goiter (61.4% vs. 38.6%), *p*-value < 0.001. The recurrent group had a higher frequency of adenopathy (94.3% vs. 5.7%), especially bilaterally (*p*-value < 0.001). The pathology results showed that the recurrent group had a greater prevalence of papillary carcinoma (55.4% vs. 44.6%), with a p-value of less than 0.001. The recurrent group exhibited considerably larger multifocality (74.4% vs. 25.6%), with a *p*-value of less than 0.001. *P*-value < 0.001 indicates that there was no high-risk classification in the non-recurrent group and 100% in the recurrent group. Higher proportions of advanced stages (T3a, T4a) were found in the tumor staging in the recurrent group (*p*-value < 0.001). The recurrent group had a considerably larger nodal involvement (N1b) (90.3% vs. 9.7%), with a *p*-value of less than 0.001. 100% of the recurrent group and 0% of the non-recurrent group experienced distant metastases (M1); the *p*-value was less than 0.001. The recurrent group had a considerably higher prevalence of advanced stage (II-IVB), with a *p*-value of less than 0.001. The response to treatment showed a stark contrast, with 99.2% of the recurrent group having a structurally incomplete response compared to 0.8% in the non-recurrent group (*p*-value < 0.001) (Table 2).

**TABLE 2:** Statistical analysis results regarding variables between the groups.

Variable	Category	Recurred				*p*-value
		No (*n* = 275)		Yes (*n* = 275)		
		*n*	%	*n*	%	
Smoking	No	259	56.1%	203	43.9%	<0.001
	Yes	16	18.2%	72	81.8%	
Hx Smoking	No	261	50.4%	257	49.6%	0.530
	Yes	14	43.8%	18	56.3%	
Hx Radiothreapy	No	274	50.5%	269	49.5%	0.466
	Yes	1	14.3%	6	85.7%	
Thyroid Function	Clinical hyperthyroidism	17	85.0%	3	15.0%	<0.001
	Clinical hypothyroidism	10	83.3%	2	16.7%	
	Euthyroid	234	46.9%	265	53.1%	
	Subclinical hyperthyroidism	5	100.0%	0	0.0%	
	Subclinical hypothyroidism	9	64.3%	5	35.7%	
Physical Examination	Diffuse goiter	7	100.0%	0	0.0%	<0.001
	Multinodular goiter	88	38.6%	140	61.4%	
	Normal	5	71.4%	2	28.6%	
	Single nodular goiter-left	63	53.4%	55	46.6%	
	Single nodular goiter-right	112	58.9%	78	41.1%	
Adenopathy	Bilateral	5	5.7%	83	94.3%	<0.001
	Extensive	0	0.0%	8	100.0%	
	Left	5	15.2%	28	84.8%	
	No	247	78.9%	66	21.1%	
	Posterior	0	0.0%	3	100.0%	
	Right	18	17.1%	87	82.9%	
Pathology	Follicular	16	42.1%	22	57.9%	<0.001
	Hurthel cell	14	63.6%	8	36.4%	
	Micropapillary	48	100.0%	0	0.0%	
	Papillary	197	44.6%	245	55.4%	
Focality	Multi-Focal	66	25.6%	192	74.4%	<0.001
	Uni-Focal	209	71.6%	83	28.4%	
Risk	High	0	0.0%	71	100.0%	<0.001
	Intermediate	38	17.8%	175	82.2%	
	Low	237	89.1%	29	10.9%	
T	T1a	48	98.0%	1	2.0%	<0.001
	T1b	38	88.4%	5	11.6%	
	T2	131	73.2%	48	26.8%	
	T3a	55	29.1%	134	70.9%	
	T3b	2	8.7%	21	91.3%	
	T4a	1	2.1%	47	97.9%	
	T4b	0	0.0%	19	100.0%	
N	N0	241	80.1%	60	19.9%	<0.001
	N1a	12	54.5%	10	45.5%	
	N1b	22	9.7%	205	90.3%	
M	M0	275	54.1%	233	45.9%	<0.001
	M1	0	0.0%	42	100.0%	
Stage	I	268	60.9%	172	39.1%	<0.001
	II	7	9.3%	68	90.7%	
	III	0	0.0%	7	100.0%	
	IVA	0	0.0%	3	100.0%	
	IVB	0	0.0%	25	100.0%	
Response	Biochemical incomplete	12	50.0%	12	50.0%	<0.001
	Excellent	207	99.5%	1	0.5%	
	Indeterminate	54	84.4%	10	15.6%	
	Structural incomplete	2	0.8%	252	99.2%	

Table 3 tabulates the results of the logistic regression analysis to determine independent biomarkers of thyroid cancer recurrence. The study employed logistic regression analysis to investigate the relevant factors that either increase or decrease the chance of DTC recurrence. The findings of this analysis are displayed in Table 3. The recurrence of thyroid cancer was significantly predicted by logistic regression analysis. Individuals with structural partial response had a significantly higher risk of recurrence (OR = 230.041, 95% CI [29.879–5299.832], *p* < 0.001), but patients with Hurthel cell pathology exhibited a significantly lower risk (OR = 0.02, 95% CI [0.001–0.25], *p* = 0.006). While indeterminate response (OR = 0.084, 95% CI [0.012–0.428], *p* = 0.005) and excellent response (OR = 0.004, 95% CI [0–0.042], *p* < 0.001) were linked to lower risk, clinical hypothyroidism was associated with reduced recurrence risk (OR = 130.849, 95% CI [2.606–8344.448], *p* = 0.014). On the other hand, a higher chance of recurrence was linked to Stage II cancer (OR = 18.593, 95% CI [3.141–146.542], *p* = 0.002) and euthyroid condition (OR = 17.419, 95% CI [1.542–394.084], *p* = 0.036). The Hosmer-Lemeshow test indicated that the model fit was adequate (χ^2^ = 0.862, df = 8, *p* = 0.999), and the model significantly predicted outcomes (χ^2^ = 695.186, df = 9, *p* < 0.001). These findings highlight the significance of thyroid function, cancer stage, pathology type, and treatment response in predicting the recurrence of thyroid cancer (Table 3).

**TABLE 3:** Results of logistic regression analysis to determine independent biomarkers of thyroid cancer recurrence.

Variables	Coef.	SE	*z*-statistic	*p*	OR	CI	Comment	ES
pathology-Hurthel cell	−3.908	1.429	−2.735	0.006	0.02	0.001–0.25	Reducing effect	Low
response -Structural Incomplete	5.438	1.228	4.43	< 0.001	230.041	29.879–5299.83	Increasing effect	High
thyroid function-Clinical Hypothyroidism	4.874	1.986	2.454	0.014	130.849	2.606–8344.44	Reducing effect	High
response-Excellent	−5.42	1.359	−3.987	< 0.001	0.004	0–0.042	Reducing effect	Low
response-Indeterminate	−2.473	0.887	−2.789	0.005	0.084	0.012–0.428	Reducing effect	Low
stage-II	2.923	0.966	3.027	0.002	18.593	3.141–146.542	Increasing effect	High
thyroid function-Euthyroid	2.858	1.364	2.096	0.036	17.419	1.542–394.084	Increasing effect	High
**Hosmer–Lemeshow test**			**The goodness of fit statistics**			**Omnibus test**		
**Chi-square statistics**	**df**	***p***	**McFadden *R*^2^**	**Cox ve Snell *R*^2^**	**Nagelkerke *R*^2^**	**Chi-square statistics**	**df**	***p***
0.862	8	0.999	0.912	0.717	0.957	695.186	9	<0.001

Coef., Coefficient; SE, Standard Error; OR, Odds Ratio; CI, Confidence Interval; ES, Effect Size; df, Degrees of Freedom.

Table 4 demonstrates the performance results of classifiers based on association rules regarding thyroid cancer recurrence prediction. The study used accuracy, sensitivity, specificity, PPV, NPV, and F1 score to compare how well two rule-based classification methods—RCAR and CBAR—knew how to predict thyroid cancer recurrence. RCAR demonstrated slightly higher accuracy (96.7%) compared to CBAR (96.0%) and also outperformed CBAR in sensitivity (94.9% vs. 93.1%), indicating its superior ability to correctly identify true positives. The F1 score, balancing both precision and recall, was 96.7% for RCAR and 95.9% for CBAR, further confirming RCAR’s better overall classification performance (Table 4).

**TABLE 4:** Performance results of classifiers based on association rules regarding thyroid cancer recurrence prediction.

Metric	RCAR	CBAR
Accuracy	0.967	0.96
Sensitivity	0.949	0.931
Specificity	0.985	0.989
Positive predictive value	0.985	0.988
Negative predictive value	0.951	0.935
F1-score	0.967	0.959

Table 5 contains organized class association rules to reveal important patterns of thyroid cancer recurrence. Papillary thyroid cancer was the most common histologic subtype, accounting for 75% of cases, followed by Hurthle cell carcinoma (12%), follicular carcinoma (7%), and micropapillary thyroid cancer (6%). Over half of the patients (54%) responded well to treatment, while 27% had structurally incomplete responses, 17% had undetermined responses, and 6% had chemically incomplete responses. Recurrence occurred in 28% of patients, with a strong association between papillary pathology with structural incomplete response and recurrence (support: 41.1%, confidence: 100%). Nodal involvement classified as N1b was another key predictor of recurrence (support: 36.0%, confidence: 100%). Conversely, the absence of adenopathy and an excellent treatment response indicated a high probability of being recurrence-free (support: 35.1%, confidence: 100%).

**TABLE 5:** The generated association rules based on the RCAR method.

Left-hand side rules	Right-hand side rules	Support	Confidence	Frequency
[Pathology = Papillary, response = Structural Incomplete]	[Recurred = Yes]	0.411	1	226
[*n* = N1b, response = Structural Incomplete]	[Recurred = Yes]	0.36	1	198
[Adenopathy = No, response = Excellent]	[Recurred = No]	0.351	1	193
[Risk = Low, response = Excellent]	[Recurred = No]	0.345	1	190
[Smoking = No, thyroid function = Euthyroid, response = Structural Incomplete]	[recurred = Yes]	0.333	1	183
[thyroid function = Euthyroid, risk = Intermediate, response = Structural Incomplete]	[Recurred = Yes]	0.28	1	154
[Thyroid function = Euthyroid, physical examination = Multinodular goiter, response = Structural Incomplete]	[Recurred = Yes]	0.222	1	122
[Thyroid function = Euthyroid, response = Structural Incomplete]	[Recurred = Yes]	0.445	0.996	245
[Response = Excellent]	[Recurred = No]	0.376	0.995	207
[Smoking = No, response = Structural Incomplete]	[Recurred = Yes]	0.336	0.995	185
[Risk = Intermediate, response = Structural Incomplete]	[Recurred = Yes]	0.287	0.994	158
[Physical examination = Multinodular goiter, response = Structural Incomplete]	[Recurred = Yes]	0.231	0.992	127
[Age = [15,52.5), gender = F, smoking = No, risk = Low]	[Recurred = No]	0.338	0.959	186
[Thyroid function = Euthyroid, focality = Multi-Focal, risk = Intermediate, *n* = N1b]	[Recurred = Yes]	0.202	0.957	111

Patients classified as low risk with excellent response also demonstrated a high likelihood of being recurrence-free (support: 34.5%). However, individuals with structurally incomplete responses, even with normal thyroid function (euthyroid), were likely to experience recurrence. This was particularly evident in intermediate-risk patients with structurally incomplete responses (Rule 6) and those with multinodular goiter despite euthyroid status (Rule 7). The most frequent rule identified involved euthyroid patients with structurally incomplete responses, indicating that treatment response is a critical factor in recurrence risk, regardless of thyroid function status (Table 5).

## 4 Discussion

The present study evaluated the performance of two rule-based classification algorithms, RCAR and CBAR, in predicting thyroid cancer recurrence. The achieved results demonstrated that RCAR outperformed CBAR in terms of accuracy (96.7% vs. 96.0%), sensitivity (94.9% vs. 93.1%), and F1 score (96.7% vs. 95.9%). These findings are consistent with previous research by Azmi et al. (9) and Zhang and Zhou (22), who have reported the superiority of RCAR over other rule-based algorithms in various classification tasks. The remarkable accuracy and sensitivity shown by RCAR in this research are especially notable since they signify the algorithm’s capacity to precisely categorize instances and detect genuine positives (cases that have recurred). In the context of thyroid cancer, rapid diagnosis of recurrence is of utmost importance for prompt management and improved patient outcomes (23).

The multiple logistic regression analysis reveals noteworthy autonomous indicators for the recurrence of thyroid cancer, which is consistent with the findings of earlier investigations. The presence of Hurthle cell pathology is related to a significantly reduced risk of recurrence (odds ratio = 0.02, 95% confidence interval: 0.001–0.25, *p* = 0.006), as reported by clinical research. A relevant medical study also found a decreased risk of recurrence associated with Hurthle cells (24). Hurthle cell thyroid carcinoma accounts for < 5% of all differentiated thyroid malignancies and is of follicular cell origin, and its biological behavior differs from other thyroid cancer histologies. Although it is considered a variant of follicular carcinoma, its different oncogenic expression may explain its different clinical behavior (25). The chance of recurrence is much higher for people who have a structural partial response (OR = 230.041, 95% CI: 29.879–5299.83, *p* < 0.001), which supports the idea that the first therapeutic response is important for predicting the outcome (26). The presence of clinical hypothyroidism greatly increases the likelihood of recurrence (odds ratio = 130.849, 95% confidence interval: 2.606–8344.44, *p* = 0.014). A high-quality response significantly decreases the likelihood of recurrence (odds ratio = 0.004, 95% confidence interval: 0–0.042, *p* < 0.001), supporting the findings of a relevant paper (5). Stage II thyroid cancer and euthyroid state are substantial risk factors for thyroid carcinoma, which are consistent with the study (27). The model’s resilience is validated using the Hosmer-Lemeshow and Omnibus tests, demonstrating a reliable match.

The association rules generated by RCAR provided valuable insights into the factors associated with thyroid cancer recurrence. For instance, the presence of papillary pathology with structural incomplete response (Rule 1) and nodal involvement with structural incomplete response (Rule 2) were strongly associated with recurrence, with 100% confidence and high support values (41.1% and 36.0%, respectively). These findings corroborate previous studies (5, 28) that have identified structural incomplete response and nodal status as significant predictors of thyroid cancer recurrence. Clinically, these rules could aid in identifying high-risk patients who may benefit from more aggressive treatment and closer monitoring (5). The aggressive PTC subtypes include the tall cell, columnar cell, diffuse sclerosing variant, and hobnail variant. Higher rates of nodal and distant metastases, multifocality, extra-thyroidal extension, recurrence, and resistance to radioactive iodine therapy have all been linked to these variations (2). It is not known exactly how many of the patients with papillary carcinoma in this study were classified as unconventional variants.

The lack of adenopathy and a good response to treatment (Rule 3) were strongly linked to freedom from recurrence. This suggests that the current methods for risk stratification are working well at finding low-risk patients. Clinical decision-making could use this information, potentially reducing the intensity of follow-up and treatment for low-risk patients (24). Unilateral or bilateral modified lymph node dissection is recommended to reduce the possibility of recurrence, especially in patients with tumor sizes greater than T2. The importance of lymphadenopathy in the disease should also be evaluated in this respect (29).

Additionally, the association between euthyroid status, constitutively incomplete response, and recurrence (Rules 5, 6, and 8) highlights the importance of considering both structural response and thyroid function in predicting recurrence, as reported by previous studies (5, 30). The importance of TSH suppression after surgery in patients with thyroid carcinoma is also known. thyroid hormone levels have long been known to be associated with recurrence. For low-risk thyroid cancer, the 2015 ATA guidelines advise keeping the TSH level between 0.5 and 2 mIU/L following thyroid lobectomy. The need for TSH suppression treatment following a lobectomy can be predicted by the preoperative TSH level. This could be useful in identifying patients who may benefit from thyroid hormone supplements. In a study, recurrence was found in 4 patients (1.1%) who did not receive any T4 supplementation and had TSH levels > 2 mIU/L after lobectomy. This situation shows us that preoperative and postoperative thyroid hormone levels are effective in recurrence. In our study, it is also shown to be important and a component that increases the risk (31). These findings suggest that even patients with normal thyroid function may require closer monitoring and potentially more aggressive treatment if they exhibit a structurally incomplete response.

It is noteworthy that the findings of this study are consistent with those reported in similar research articles cited in the text. Research papers (9, 32) also found that rule-based algorithms, particularly RCAR, outperformed other classification methods in various domains. Additionally, RCAR’s association rules align with the findings of research studies (33, 34) that examined the significance of factors such as pathology, nodal status, structural response, and thyroid function in predicting the recurrence of thyroid cancer.

The findings of this study have several clinical implications for the management of thyroid cancer patients. The high accuracy and sensitivity of the RCAR algorithm in predicting recurrence suggest that it could be integrated into clinical decision support systems to aid in risk stratification and treatment planning (35). The association rules generated by RCAR could be used to identify high-risk patients who may benefit from more aggressive treatment and closer monitoring, as well as low-risk patients who may be candidates for less intensive follow-up and treatment (5, 24).

Furthermore, the identification of specific factors associated with recurrence, such as pathology, nodal status, structural response, and thyroid function, reinforces the importance of considering these variables in risk assessment and clinical decision-making. This information could be used to develop more personalized treatment plans and follow-up strategies based on individual patient characteristics (30). The association rules obtained in the study can also guide us in the clinical decision-making process. In particular, factors such as papillary pathology, nodal involvement, and inadequate structural response appear to have a strong association with recurrence (Rules 1–2). Such high-risk patients may require more aggressive treatment regimens and closer follow-up (5).

On the other hand, the association of excellent structural response and absence of adenopathy with a low risk of recurrence (Rule 3) suggests that a less intensive follow-up and treatment plan may be appropriate for this patient group (36). Proper application of risk stratification systems can help us avoid unnecessary intensive treatment and follow-up. The association of structural incomplete response with recurrence even in euthyroid patients (Rules 5–8) highlights the need to consider structural response in addition to thyroid function tests (30). This finding suggests that some patients may require close monitoring or more aggressive treatment despite having normal thyroid function.

However, it is important to note that the clinical implementation of these findings should be evaluated carefully. While the RCAR algorithm and the association rules demonstrate promising performance, further validation on larger and more diverse datasets may be necessary before widespread adoption in clinical practice. Additionally, the interpretability and transparency of the rule-based models should be carefully evaluated to ensure that the decisions made based on these models are explainable and accountable (37).

A structurally incomplete response is a major risk factor that greatly increases the risk under all conditions. It is known that as the size of the thyroid tumor increases and the stage shifts towards the advanced stage, the risk of recurrence and the prognosis of the disease will be worse. However, due to the relatively small distribution of patients in this stratification, our methods were unable to detect a significant association. A general rule of thumb is that tumor size and stage are associated with a worse prognosis (38, 39).

In general, differentiated thyroid carcinomas have a good prognosis, but it is critical to identify those patients at risk of progressive disease with a poor clinical course and those at high risk of death at the time of diagnosis. Recognizing prognostic factors and being able to figure out the risk of recurrence are important for the best management of DTC. So are the extent of thyroid surgery and the reasons for radioiodine therapy after surgery. Methods to facilitate the determination of the risk of recurrence are critical in groups where a poor clinical outcome is expected (39). This study has a few limitations. Firstly, the analysis may not use a representative dataset of all thyroid cancer cases, and the results may differ when applied to different populations or clinical settings. Secondly, the study focused solely on rule-based classification algorithms, and it would be valuable to compare their performance with other machine learning techniques, such as neural networks or ensemble methods.

In conclusion, the current study demonstrated the efficacy of the RCAR algorithm in predicting thyroid cancer recurrence and provided valuable insights into the factors associated with recurrence through the generated association rules. The findings have potential clinical implications for risk stratification, treatment planning, and follow-up strategies in thyroid cancer management. However, further evaluation and validation are necessary before widespread clinical implementation.

## Data Availability

The original contributions presented in the study are included in the article/supplementary material, further inquiries can be directed to the corresponding authors.
